# Lycopene, Lutein and Zeaxanthin May Reduce Faecal Blood, Mucus and Pus but not Abdominal Pain in Individuals with Ulcerative Colitis

**DOI:** 10.3390/nu8100613

**Published:** 2016-09-30

**Authors:** Dominika Głąbska, Dominika Guzek, Paulina Zakrzewska, Dariusz Włodarek, Gustaw Lech

**Affiliations:** 1 Department of Dietetics, Faculty of Human Nutrition and Consumer Sciences, Warsaw University of Life Sciences (WULS-SGGW), 159c Nowoursynowska Str., 02-776 Warsaw, Poland; paulina_zakrzewska@sggw.pl (P.Z.); dariusz_wlodarek@sggw.pl (D.W.); 2 Department of Organization and Consumption Economics, Faculty of Human Nutrition and Consumer Sciences, Warsaw University of Life Sciences (WULS-SGGW), 159c Nowoursynowska Str., 02-776 Warsaw, Poland; dominika_guzek@sggw.pl; 3 Department of General, Gastroenterological and Oncological Surgery, Medical University of Warsaw, 1a Banacha Str., 02-097 Warsaw, Poland; gustaw.lech@wum.edu.pl

**Keywords:** ulcerative colitis, vitamin A, lycopene, lutein and zeaxanthin, faecal blood, faecal mucus

## Abstract

Background: The main symptom of ulcerative colitis is diarrhoea, which is often accompanied by painful tenesmus and faecal blood and mucus. It sometimes co-occurs with abdominal pain, fever, feeling of fatigue, loss of appetite and weight loss. Some dietary factors have been indicated as important in the treatment of ulcerative colitis. The aim of the study was to analyse the association between retinoid intake (total vitamin A, retinol, β-carotene, α-carotene, β-cryptoxanthin, lycopene, lutein and zeaxanthin) and ulcerative colitis symptoms (abdominal pain, faecal blood, faecal mucus, faecal pus) in individuals with ulcerative colitis in remission. Methods: Assessment of diet was based on self-reported data from each patient’s dietary records taken over a period of three typical, random days (2 weekdays and 1 day of the weekend). Results: A total of 56 individuals with ulcerative colitis in remission (19 males and 37 females) were recruited for the study. One in every four individuals with ulcerative colitis in remission was characterised as having inadequate vitamin A intake. Higher lycopene, lutein and zeaxanthin intakes in individuals with ulcerative colitis in remission were associated with lower faecal blood, mucus and pus but not with lower incidence of abdominal pain. Higher carotene intake in individuals with ulcerative colitis in remission may contribute to higher incidence of faecal mucus. Conclusions: Optimising intake of specific retinoids may enhance disease control in individuals with ulcerative colitis. Prospective studies, including patient reported and objective outcomes, are required to confirm this.

## 1. Introduction

The main symptom of ulcerative colitis is diarrhoea, which is often accompanied by painful tenesmus and faecal blood and mucus. It sometimes co-occurs with abdominal pain, fever, feeling of fatigue, loss of appetite and weight loss; with its intensity depending on the activity and location of the disease [1]. The presence of diarrhoea is associated with impairment of the colon’s specialised functions, such as absorption of water and minerals, and stool formation [2].

Medical treatment in ulcerative colitis is aimed at relieving such symptoms as diarrhoea, faecal blood and abdominal pain as well as at preventing exacerbation and disease complications [3]. The choice of treatment method depends on the main symptoms and severity of the disease, and diet therapy is applied in approximately 10% of individuals with ulcerative colitis in Western countries [4], even though it is not a method of proven effectiveness [5,6]. However, some dietary factors have been indicated as important not only in the etiology of ulcerative colitis but also in treatment, which is associated with their important potential for relieving symptoms and prolonging remission [7]. However, no precise dietetic recommendations have been formulated as no explicit results have been published so far.

Among the factors analysed in the context of the course of ulcerative colitis and its symptoms is retinoid intake, but so far little is known about the existing associations. In the research of Urbano et al. [8], it was found that retinoid intake did not differ among groups of individuals with distal colitis, left colitis and pancolitis, and such results were observed for vitamin A, β-carotene, α-carotene, β-cryptoxanthin, lycopene as well as lutein and zeaxanthin. On the other hand, in the study of Ianco et al. [9], multiple differences were observed when individuals with recurrent acute and chronic pouchitis were compared to normal pouch patients. Individuals with recurrent acute and chronic pouchitis were characterised as having significantly lower intake of vitamin A, cryptoxanthin and lycopene, and for β-carotene a close-to-significant difference was observed. Moreover, the authors of the above-mentioned study [9] suggested that the intake of specific anti-oxidative nutrients, such as retinoid, might not only prevent further inflammation in individuals with ulcerative colitis but may also possibly reverse it.

The aim of the study was to analyse the association between retinoid intake (total vitamin A, retinol, β-carotene, α-carotene, β-cryptoxanthin, lycopene, lutein and zeaxanthin) and ulcerative colitis symptoms (abdominal pain, faecal blood, faecal mucus, faecal pus) in individuals in remission. The study’s hypothesis was that retinoid intake may influence gastrointestinal symptoms of ulcerative colitis, such as abdominal pain, faecal blood, faecal mucus and faecal pus in individuals in remission.

## 2. Materials and Methods

### 2.1. Study Design

The study was conducted at the Dietetic Outpatient Clinic of the Department of Dietetics, Warsaw University of Life Sciences (WULS-SGGW). Three-day dietary records of individuals with ulcerative colitis in remission were analysed. The study was conducted according to the guidelines laid down in the Declaration of Helsinki and all procedures involving human subjects were approved by the Bioethical Commission of the Central Clinical Hospital of the Ministry of Interior in Warsaw (No. 35/2009) and by the Bioethical Commission of the National Food and Nutrition Institute in Warsaw (No. 1604/2009).

### 2.2. Study Participants

The study was conducted among individuals with ulcerative colitis in remission, both males and females who were recruited and monitored at the following Warsaw Gastroenterology Outpatient Clinics: *Gastroenterology* Outpatient Clinic at Maria Skłodowska-Curie Memorial Cancer Centre, Institute of Oncology; *Gastroenterology* Outpatient Clinic at the Central Clinical Hospital of the Ministry of Interior in Warsaw and *Gastroenterology* Outpatient Clinic at the Public Central Teaching Hospital in Warsaw.

A total of 56 individuals with ulcerative colitis in remission (19 males and 37 females) were recruited for the study. Inclusion criteria were: Free-living patients with endoscopically diagnosed ulcerative colitis and confirmed clinical remission (assessed on the basis of the Mayo Scoring System and the Rachmilewitz index for assessment of ulcerative colitis activity), accompanied by confirmed endoscopic remission (image with no changes or disappearance of the vascular network, erythema, inflammatory polyps allowed) if routine endoscopy was conducted during last 6 weeks, age 18–80 years, individuals characterised by clinical remission lasting at least 6 weeks, and a constant dose of drugs for at least 6 weeks. All of the participants provided written consent to participate in the study.

All of the participants with ulcerative colitis in remission were interviewed and asked about the presence of abdominal pain, faecal blood, faecal mucus and faecal pus. They were asked to compare the current frequency and intensity of the symptoms with those before the diagnosis of ulcerative colitis. A lack of abdominal pain, faecal blood, faecal mucus and faecal pus during remission or no increase in frequency or intensity of symptoms compared to those observed before the ulcerative colitis diagnosis were interpreted as a lack of the above-mentioned symptoms.

### 2.3. Analysis of Diet

The assessment of diet was based on self-reported data from the patients’ dietary records conducted over a period of three typical, random days (2 weekdays and 1 day of the weekend). The dietary record was conducted on the basis of widely accepted and applied rules [10]. To provide reliable evaluations of food intake, the participants were instructed about the principles of dietary record and about the necessity of conducting an accurate and scrupulous record of all consumed food products and beverages, whereas the serving sizes were verified by using the Polish Atlas of Food Products’ and Dishes’ Portion Sizes [11]. The energy values of the diets were analysed using Dietetyk Polish dietician software version 2.0 and the Polish base of the nutritional values of products [12]. The intakes of retinoids: Total vitamin A (µg retinol activity equivalent), retinol (µg), β-carotene (µg), α-carotene (µg), β-cryptoxanthin (µg), lycopene (µg), lutein and zeaxanthin (µg) were assessed by using the National Nutrient Database for Standard Reference of the United States Department of Agriculture [13] due to the lack of a Polish database. The obtained average intakes of the analysed nutrients (mean value of three recorded days) constituted the basis for further analysis. The retinoid intake levels were presented in µg and were recalculated per 1000 kcal of diet (µg/1000 kcal) in order to also analyse the nutrient density of the diet. Retinoid intakes were compared among sub-groups of individuals affected and unaffected by ulcerative colitis symptoms, i.e., groups of individuals affected by specified ulcerative colitis symptoms (abdominal pain, faecal blood, faecal mucus, faecal pus) were treated as the analysed groups and those groups of individuals unaffected by symptoms were treated as control groups.

No specific recommendations for vitamin A have been formulated for individuals with inflammatory bowel disease. The total content of vitamin A in diets was compared with recommendations for healthy men and women at the Estimated Average Requirement (EAR) level, i.e., a 630 µg retinol activity equivalent for males and a 500 µg retinol activity equivalent for females [14] since the recommendations have not been specified for other retinoids (retinol, ecoarotene, α-carotene, β-cryptoxanthin, lycopene, lutein and zeaxanthin).

### 2.4. Statistical Analysis

The obtained data are presented as means ± standard deviations (SD) with minimum, maximum and median values. The distributions of the analysed factors were verified by using the Shapiro-Wilk test. Differences between groups were identified by using the U Mann-Whitney test (applied for non-parametric distribution). The accepted level of significance was set at *p* ≤ 0.1. Statistical analysis was conducted using Statistica software version 8.0 (StatSoft Inc., Tulsa, OK, USA).

## 3. Results

Vitamin A intake for the majority of individuals with ulcerative colitis (73%) was at the recommended level, however, for some individuals it was significantly lower than recommended, as in general it varied from 291.5 µg to 16,464.4 µg.

### 3.1. Retinoid Intake in Individuals with Ulcerative Colitis Declaring Either a Lack or Presence of Abdominal Pain

The levels of retinoid intake in groups of individuals with ulcerative colitis declaring either a lack or presence of abdominal pain are presented in Table 1. No differences were observed in retinoid intake between groups.

### 3.2. Retinoid Intake in Individuals with Ulcerative Colitis Declaring Either a Lack or Presence of Faecal Blood

The levels of retinoid intake in groups of individuals with ulcerative colitis who declared either a lack or presence of faecal blood are presented in Table 2. Higher lycopene intake was stated by individuals declaring a lack of faecal blood (median 2496.3 µg) in comparison with those who had declared the presence of faecal blood (median 1438.3 µg) (*p* = 0.064).

### 3.3. Retinoid Intake in Individuals with Ulcerative Colitis Declaring Either a Lack or Presence of Faecal Mucus

The levels of retinoid intake in groups of individuals with ulcerative colitis who declared either a lack or presence of faecal mucus are presented in Table 3. The number of individuals affected by faecal mucus was rather small; it was stated by 16% of the analysed group of ulcerative colitis patients. Higher lutein and zeaxanthin intake was stated by individuals declaring a lack of faecal mucus (median 1356.7 µg) in comparison with individuals who had declared the presence of faecal mucus (median 873.1 µg) (*p* = 0.074). The opposite relation was observed for β-carotene and α-carotene after recalculating the nutritional value per 1000 kcal. Lower intake of β-carotene (*p* = 0.040) was identified in individuals who had declared a lack of faecal mucus (median 1575.4 µg/1000 kcal) in comparison with those who had declared its presence (median 2903.0 µg/1000 kcal). Similarly, a lower intake of α-carotene (*p* = 0.071) was stated by individuals who had declared a lack of faecal mucus (median 363.1 µg/1000 kcal) in comparison to those who had declared its presence (median 1165.5 µg/1000 kcal).

### 3.4. Retinoid Intake in Individuals with Ulcerative Colitis Declaring Either a Lack or Presence of Faecal Pus

The levels of retinoid intake in groups of individuals with ulcerative colitis who had declared either a lack or presence of faecal pus are presented in Table 4. The number of individuals affected by faecal pus was rather small; it was stated by 5% of the analysed group of ulcerative colitis patients. Higher lutein and zeaxanthin intake was stated by individuals who had declared a lack of faecal pus (median 1320.6 µg), in comparison with those who had declared its presence (median 540.6 µg) (*p* = 0.038). The observed significance was also indicated (*p* = 0.063) after recalculating the nutritional value per 1000 kcal, as individuals who had declared a lack of faecal pus were still characterised by higher lutein and zeaxanthin intake (median 723.7 µg/1000 kcal) than those individuals who had declared its presence (median 395.5 µg/1000 kcal).

## 4. Discussion

Inadequate intake of vitamin A is quite common among individuals with inflammatory bowel disease. In the research of other authors, vitamin A intakes lower than the recommended level were observed in the case of 18% [15] to 40% of individuals with ulcerative colitis [8], and in 21% [16] to 69% [17] of those with Crohn’s disease.

In the presented study, it was decided to include only patients with ulcerative colitis and not with Crohn’s disease, as not only do the gastrointestinal symptoms differ in these two conditions [18] but also because the effects of retinoid intake may differ. This was already stated in a study on 13-*cis*-retinoic acid (isotretinoin) [19]. Moreover, it was decided to conduct an analysis only on individuals in remission, as the diet should be changed to a low-fibre one during the exacerbation period [20], thus retinoid intake in such a diet is also lower [21]. This was observed in the study of da Silva et al. [22], as retinol intake in individuals with Crohn’s disease in remission was higher than during exacerbation. As there is no international agreement on the scoring system used to measure ulcerative colitis activity [23], the Mayo Scoring System was chosen since it has been indicated as the best-known disease activity instrument for ulcerative colitis [24], and the Rachmilewitz index was chosen as an additional method to confirm the results by using two methods independently instead of only one. Although diarrhoea and the presence of faecal blood are typically symptoms of the exacerbation period and not of remission [23], defined remission does not exclude their presence [25]. In the Mayo Scoring System [26] and Rachmilewitz index, both of which serve to assess ulcerative colitis activity [27], diarrhoea and faecal blood do not determine the exacerbation periods.

In the analysed group of individuals with ulcerative colitis, the observed share of patients characterised by inadequate intake of vitamin A was similar as in the above-mentioned research studies. On the basis of these studies, it may be stated that at least one in every four individuals with ulcerative colitis does not have a properly balanced diet and has inadequate vitamin A intake.

Vitamin A intake levels in individuals with inflammatory bowel disease is lower not only than the recommended values but also than values observed for healthy individuals, as was proven for individuals with ulcerative colitis [28] and individuals after pouch surgery [9].

Low vitamin A intake generates deficiencies that are identified quite commonly in individuals with paediatric inflammatory bowel disease [29], especially in newly diagnosed patients [30]. The blood retinoid level in patients with inflammatory bowel disease is commonly significantly lower than in healthy individuals, as total vitamin A and β-carotene blood levels were observed to be significantly lower in affected individuals [31,32]. Similarly, in the group of patients with Crohn’s disease, total vitamin A, β-carotene, α-carotene, lutein and zeaxanthin levels in the blood were lower than for healthy individuals [33]. In the analysed group of individuals with ulcerative colitis, if not only vitamin A intake but also vitamin A blood levels were lower than the reference values, such a result may suggest a deficiency of this nutrient.

However, it should be noted that even if total vitamin A intake did not differ among individuals after pouch surgery and the healthy control individuals, lower β-carotene, α-carotene and lycopene blood levels were observed in patients with a pouch [34]. As a consequence, it might be concluded that vitamin A deficiency in individuals with inflammatory bowel disease is caused not only by lower vitamin intake but also by other factors. The first possible factor that may play an important role is impaired fat digestion and lower fat absorption, as has been stated for inflammatory bowel disease [35,36], thus resulting in lower absorption of retinoids which are fat-soluble compounds. The other possible reason is the higher requirement for anti-oxidative compounds as observed in individuals with inflammatory bowel disease that may result from increased oxidative stress [37] causing enhanced turnover [34].

The decreased antioxidant defence that occurs due to insufficient antioxidant intake may have an influence on the intestine by inducing higher tissue susceptibility to oxidative damage and by hindering both recovery of the mucosa and improvement of the epithelial cell layer integrity, as has been observed in individuals with inflammatory bowel disease [38]. It has been confirmed so far that dietary vitamin A and vitamin E have the potential to scavenge free radicals in individuals after pouch surgery [39].

The oxidative injury that has been observed in individuals with inflammatory bowel disease, caused by a possible dietary influence and overproduction of colonic oxidants, is correlated with disease severity [40]. Such a specific association was observed for vitamin A in the blood level. The significant link between vitamin A hypovitaminosis and disease activity was revealed in Crohn’s disease-affected children [30] and adults [41]. Moreover, the results of previous Polish studies indicated that in inflammatory bowel disease-affected individuals, serum retinol levels were inversely correlated with clinical activity of the disease [42]. It was observed that the lowest values of serum retinol level were seen in the case of patients with severe and moderately severe ulcerative colitis, while values stated for mild ulcerative colitis individuals and remission individuals were comparable with controls [43].

In the research of Urbano et al. [8] and Ianco et al. [9], the conducted analyses were similar to those in the study presented here and vitamin A intakes, not vitamin A blood levels, were assessed. However, in the above-mentioned research it was not the defined symptoms but the extent of the injury [8] and pouch status [9] that were assessed. The results of the above-mentioned studies were ambiguous. Even though the retinoid intakes were not associated with the extent of injury [8], they were associated with pouch status [9].

The observations regarding lycopene, lutein and zeaxanthin made in the analysis presented here confirm the general observations of Ianco et al. [9]. However, in the current study it was not the general course of the disease that was taken into account but rather the detailed symptoms observed in the remission. It might be suggested that faecal blood, mucus and pus are especially prone to be reduced due to higher lycopene, lutein and zeaxanthin intake, while such an influence was not observed for abdominal pain. Simultaneously, in the research of Ianco et al. [9], lutein and zeaxanthin intakes were not analysed, although a similar influence of lycopene as in the presented study was indicated. Therefore, it may be concluded that both lycopene and lutein with zeaxanthin are potential nutrients relieving ulcerative colitis symptoms. This may be explained by the fact that both lycopene [44] and lutein [45] are powerful antioxidants and a wide range of possible beneficial effects has been well documented in both cases.

The diet has been analysed in studies as a potential etiologic [46] and therapeutic factor [47] for treating ulcerative colitis. However, so far only a small number of research studies have been published indicating a proven association between diet and ulcerative colitis incidence or course [6]. In the case of retinoids, no effect of retinol and carotene intake on the risk of developing ulcerative colitis was identified in the European prospective cohort study [48]. However, it was hypothesised by Andersen et al. [49] that vitamin A acting via initiating the immune response may exert a significant influence on intestinal inflammation and protection from pathogens. It was observed that retinoic acid takes part in the translocation of antigen-presenting B and T cells to the gut and that dendritic cells have the ability to induce regulatory T cells in the presence of vitamin A metabolites, whereas without vitamin A metabolites, inflammation-promoting Th17 cells are induced [49]. Simultaneously, the abundant experimental and human observations conducted with healthy individuals and patients with gastric or duodenal ulcers clearly proves the defensive role of retinoids in the gastrointestinal tract associated with preventing chemically-induced gastric mucosal damage without inhibiting gastric acid secretion [50]. A similar possible colonic influence has also been suggested since retinoids have been taken into account as potential factors promoting gastrointestinal mucosal integrity [51].

On the other hand, apart from the significant anti-inflammatory potential of retinoids, some authors have also indicated the opposite effect, e.g., retinol was stated to be positively associated with inflammatory bowel disease risk in a study by Reif et al. [52]. Also in the animal model, two mechanisms of action of tomato lycopene extract in relation to colitis were detected. It was proven that lycopene prevents lipopolysaccharide-induced pro-inflammatory gene expression but simultaneously also aggravates dextran sulphate sodium-induced colitis [53]. The possibility of such a pro-inflammatory effect was indicated especially in the case of carotene, as β-carotene may underlie interactions with quercetin or naringenin (flavonoids) and, in consequence, it may promote the secretion of pro-inflammatory mediators [54]. The above-mentioned interactions have been observed in intestine cells and it seems that other flavonoids also have the potential to interact [55], thus such data indicate the requirement for further analysis involving the possibility of the influence of carotenoids on intestinal inflammatory processes. Moreover, since carotenoids and flavonoids are found combined in the same plant-based food products [56], it is difficult to conduct human studies analysing the influence of carotene itself without the impact of other nutrients. However, the results observed for the analysed group suggest that high β-carotene and α-carotene intake in individuals with ulcerative colitis may exert a negative influence, enhancing incidence of faecal mucus.

On the basis of the number of studies suggesting the role of retinoids in the course or activity of inflammatory bowel disease (summarised in Table 5) and the obtained unclear results, it should be noted that the influence of retinoids is not just a simple correlation. The results observed in the study presented here may partly explain retinoid action. On the other hand, it should be stated that not only do retinoids seem to alleviate some of the ulcerative colitis symptoms (other than abdominal pain), but also that some retinoids might have the opposite impact. However, the fact that in the Polish diet main sources of various retinoids are diverse [57] allows for control of dietary retinoid intake in individuals with ulcerative colitis. As a result, excessive vitamin A intake, especially from supplementation, must be indicated as not recommended, as it may be associated with numerous harmful consequences as indicated by the European Food Safety Authority (EFSA), e.g., skin and musculoskeletal system disorders, liver toxicity, or teratogenic effects [58]. In particular, in the case of individuals with inflammatory bowel disease, Hwang et al. [59] indicated that signs of vitamin A toxicity should be closely monitored.

However, the relatively small number of analysed subjects must be indicated as a limitation of the study. Due to the small number of cases, significance at the conventional cut-off value of 0.05 was not chosen and, according to the assumption by Fisher [60] that if the *p*-Value is higher than 0.1 there is certainly no reason to suspect the hypothesis tested, the less stringent level of significance at *p* ≤ 0.1 (10%) was chosen; it is also allowed [61].

Taking into account the number of subjects analysed, the number of subjects in sub-groups of individuals affected by specific symptoms of ulcerative colitis was small. This was stated especially in the case of faecal mucus and faecal pus, which were quite rare in the analysed group. As a consequence, further analysis conducted on larger groups of individuals with inflammatory bowel disease is required in order to provide not only a larger group assessment but also a higher number of individuals affected by specific symptoms.

Moreover, further studies should include not only an analysis of vitamin A intake but also an analysis of the vitamin A blood level, as it would be necessary to make conclusions about the direct influence of retinoid intake on ulcerative colitis symptoms. However, the results obtained here are promising.

## 5. Conclusions

1. One in every four individuals with ulcerative colitis in remission was characterised as having inadequate vitamin A intake.
2. Higher lycopene, lutein and zeaxanthin intakes in individuals with ulcerative colitis in remission were associated with lower faecal blood, mucus and pus but not with lower incidence of abdominal pain.
3. Higher carotene intake in individuals with ulcerative colitis in remission may contribute to higher incidence of faecal mucus.
4. Optimising intake of specific retinoids may enhance disease control in individuals with ulcerative colitis. Prospective studies, including patient reported and objective outcomes, are required to confirm this.

## Figures and Tables

**Table 1:** The levels of retinoid intake in groups of individuals with ulcerative colitis declaring either a lack or presence of abdominal pain (comparison conducted using U Mann-Whitney test).

		Individuals Declaring Lack of Abdominal Pain (*n* = 29)		Individuals Declaring Abdominal Pain (*n* = 27)		*p*-Value
		Mean ± SD	Median (min–max)	Mean ± SD	Median (min–max)	
Intake	Vitamin A (µg retinol activity equivalent)	999.5 ± 724.3	795.5 (327.1–3525.1)	1656.8 ± 3250.8	708.8 (291.5–16,464.4) *	0.793
	Retinol (µg)	471.6 ± 288.8	376.9 (119.9–1231.9)	1042.4 ± 2993.6	429.8 (120.7–15,899.8) *	0.974
	β-carotene (µg)	5358.4 ± 6218.9	2758.7 (64.5–25,850.5)	6191.8 ± 13,315.5	2964.9 (624.5–71,862.3) *	0.974
	α-carotene (µg)	1708.2 ± 2416.4	592.3 (7.5–9560.0) *	2133.1 ± 5569.7	810.3 (13.2–29,557.3) *	0.818
	β-cryptoxanthin (µg)	143.2 ± 183.9	51.9 (3.6–585.3)	66.6 ± 72.8	39.4 (1.9–289.0) *	0.431
	Lycopene (µg)	224.0 ± 2976.2	1154.7 (0.0–13,411.9)	1958.6 ± 1698.7	2182.4 (0.2–5359.9) *	0.588
	Lutein + zeaxanthin (µg)	1840.6 ± 2357.1	1320.6 (289.0–13,221.3)	1601.0 ± 1209.4	1298.6 (432.7–5323.8) *	0.909
Intake per 1000 kcal	Vitamin A (µg retinol activity equivalent/1000 kcal)	482.1 ± 430.1	364.4 (158.4–2365.9) *	891.0 ± 1815.5	371.1 (171.5–8947.0) *	0.544
	Retinol (µg/1000 kcal)	226.9 ± 148.3	181.0 (82.8–662.5) *	543.1 ± 1623.6	213.4 (79.1–8640.2) *	0.412
	β-carotene (µg/1000 kcal)	2583.7 ± 3585.3	1674.5 (61.3–17,349.9) *	3505.2 ± 8532.8	1621.6 (289.9–45,899.7) *	0.694
	α-carotene (µg/1000 kcal)	816.0 ± 1361.4	376.3 (3.5–5984.6) *	1224.6 ± 3558.5	436.9 (11.3–18,878.8) *	0.566
	β-cryptoxanthin (µg/1000 kcal)	79.6 ± 135.5	25.3 (1.0–644.0) *	36.8 ± 42.8	15.3 (1.2–183.0) *	0.634
	Lycopene (µg/1000 kcal)	997.5 ± 1183.0	633.0 (0.0–4435.3) *	1034.2 ± 952.5	1018.4 (0.1–3260.4) *	0.491
	Lutein + zeaxanthin (µg/1000 kcal)	846.5 ± 767.8	566.1 (191.9–3505.7) *	853.3 ± 627.3	724.6 (206.9–2906.1) *	0.512

* Nonparametric distribution (verified using the Shapiro-Wilk test; *p* < 0.05).

**Table 2:** The levels of retinoid intake in groups of individuals with ulcerative colitis declaring either a lack or presence of faecal blood (comparison conducted using U Mann-Whitney test).

		Individuals Declaring Lack of Faecal Blood (*n* = 17)		Individuals Declaring Faecal Blood (*n* = 39)		*p*-Value
		Mean ± SD	Median (min–max)	Mean ± SD	Median (min–max)	
Intake	Vitamin A (µg retinol activity equivalent)	915.5 ± 579.7	845.1 (329.0–2775.9) *	1491.2 ± 2743.6	681.6 (291.5–16,464.4) *	0.986
	Retinol (µg)	435.2 ± 196.0	392.7 (119.9–839.3)	882.6 ± 2496.9	359.8 (120.7–15,899.8) *	0.831
	β-carotene (µg)	4876.6 ± 5741.5	3063.4 (273.8–23,821.8) *	6145.3 ± 11,728.1	2817.5 (64.5–71,862.3) *	0.817
	α-carotene (µg)	1503.3 ± 2231.7	531.5 (7.5–9560.0) *	2091.7 ± 4836.2	759.9 (9.0–29,557.3) *	0.887
	β-cryptoxanthin (µg)	84.9 ± 140.5	44.6 (5.0–585.3) *	115.6 ± 148.7	51.9 (1.9–571.4) *	0.444
	Lycopene (µg)	2792.3 ± 2318.1	2496.3 (0.1–8317.1) *	1807.5 ± 2442.6	1438.3 (0.0–13,411.9) *	0.064
	Lutein + zeaxanthin (µg)	1836.2 ± 1181.0	1630.3 (496.2–5323.8) *	1676.7 ± 2126.2	1263.3 (289.0–13,221.3) *	0.105
Intake per 1000 kcal	Vitamin A (µg retinol activity equivalent/1000 kcal)	435.0 ± 277.8	362.0 (158.4–1411.9) *	785.7 ± 1543.4	366.8 (159.3–8947.0) *	0.708
	Retinol (µg/1000 kcal)	199.9 ± 66.8	196.9 (104.0–360.4)	457.6 ± 1354.2	187.0 (79.1–8640.2) *	0.803
	β-carotene (µg/1000 kcal)	2378.3 ± 2667.1	1934.6 (127.7–12,116.5) *	3311.2 ± 7505.6	1579.2 (61.3–45,899.7) *	0.762
	α-carotene (µg/1000 kcal)	737.2 ± 1122.9	392.8 (3.5–4862.5) *	1133.2 ± 3084.0	409.8 (8.6–18,878.8) *	0.957
	β-cryptoxanthin (µg/1000 kcal)	58.9 ± 152.5	11.8 (3.0–644.0) *	59.0 ± 75.3	23.9 (1.0–294.0) *	0.159
	Lycopene (µg/1000 kcal)	1226.0 ± 932.3	1045.7 (0.1–3564.5) *	923.3 ± 1121.5	610.6 (0.0–4435.3) *	0.130
	Lutein + zeaxanthin (µg/1000 kcal)	901.4 ± 508.5	839.8 (191.9–1808.9)	827.3 ± 770.0	630.8 (196.3–3505.7) *	0.232

* Nonparametric distribution (verified using the Shapiro-Wilk test; *p* < 0.05).

**Table 3:** The levels of retinoid intake in groups of individuals with ulcerative colitis declaring either a lack or presence of faecal mucus (comparison conducted using U Mann-Whitney test).

		Individuals Declaring Lack of Faecal Mucus (*n* = 47)		Individuals Declaring Faecal Mucus (*n* = 9)		*p*-Value
		Mean ± SD	Median (min–max)	Mean ± SD	Median (min–max)	
Intake	Vitamin A (µg retinol activity equivalent)	928.0 ± 643.7	681.6 (311.9–3525.1) *	3344.9 ± 5387.2	1076.0 (291.5–16,464.4) *	0.435
	Retinol (µg)	486.2 ± 338.9	383.5 (119.9–2070.9) *	2107.6 ± 5181.2	236.9 (132.8–15,899.9) *	0.422
	β-carotene (µg)	4507.6 ± 5059.2	2811.5 (64.5–25,850.5) *	12,301.3 ± 22,545.1	5588.6 (1122.6–71,862.3) *	0.188
	α-carotene (µg)	1338.4 ± 1943.5	711.0 (7.5–9560.0) *	4913.9 ± 9369.2	2219.4 (13.2–29,557.3) *	0.147
	β-cryptoxanthin (µg)	111.0 ± 155.4	44.6 (3.6–585.3) *	82.0 ± 79.1	53.5 (1.9–246.9)	0.789
	Lycopene (µg)	2287.5 ± 2504.4	2015.0 (0.0–13,411.9) *	1160.9 ± 1805.2	0.9 (0.2–4502.7) *	0.160
	Lutein + zeaxanthin (µg)	1816.2 ± 1964.9	1356.7 (289.0–13,221.3) *	1249.5 ± 1301.7	873.1 (432.7–4549.9) *	0.074
Intake per 1000 kcal	Vitamin A (µg retinol activity equivalent/1000 kcal)	405.5 ± 209.4	360.4 (158.4–1411.9) *	543.7 ± 454.2	512.2 (248.6–8946.9) *	0.103
	Retinol (µg/1000 kcal)	223.7 ± 131.0	190.2 (85.5–726.7) *	287.9 ± 233.1	205.2 (79.1–8640.2) *	0.964
	β-carotene (µg/1000 kcal)	1854.6 ± 1827.2	1575.4 (61.3–12,116.5) *	3347.8 ± 4040.5	2903.0 (813.8–45,899.7) *	0.040
	α-carotene (µg/1000 kcal)	530.5 ± 759.5	363.1 (3.5–4862.5) *	1342.3 ± 1659.3	1165.5 (11.3–18,878.8) *	0.071
	β-cryptoxanthin (µg/1000 kcal)	61.1 ± 111.6	15.6 (1.0–644.0) *	172.7 ± 223.3	25.5 (1.2–243.2) *	0.489
	Lycopene (µg/1000 kcal)	1025.6 ± 972.4	842.3 (0.0–3827.9) *	1918.3 ± 1667.2	1025.6 (0.0–4435.3) *	0.265
	Lutein + zeaxanthin (µg/1000 kcal)	819.7 ± 634.3	662.7 (191.9–3505.7) *	905.2 ± 1175.9	634.3 (94.5–3505.7) *	0.284

* Nonparametric distribution (verified using the Shapiro-Wilk test; *p* < 0.05).

**Table 4:** The levels of retinoid intake in groups of individuals with ulcerative colitis declaring either a lack or presence of faecal pus (comparison conducted using U Mann-Whitney test).

		Individuals Declaring Lack of Faecal Pus (*n* = 53)		Individuals Declaring Faecal Pus (*n* = 3)		*p*-Value
		Mean ± SD	Median (min–max)	Mean ± SD	Median (min–max)	
Intake	Vitamin A (µg retinol activity equivalent)	1335.3 ± 2375.3	767.6 (291.5–16,464.4) *	983.6 ± 899.3	615.1 (327.1–2008.6)	0.743
	Retinol (µg)	755.1 ± 2145.7	376.9 (119.9–15,899.8) *	600.5 ± 460.6	562.1 (160.2–1079.1)	0.662
	β-carotene (µg)	5872.4 ± 10,422.2	2964.9 (64.5–71,862.3) *	3778.1 ± 4562.6	1700.0 (624.5–9009.7)	0.423
	α-carotene (µg)	1938.8 ± 4299.7	759.9 (7.5–29,557.3) *	1457.6 ± 2299.6	194.4 (66.4–4111.9)	0.560
	β-cryptoxanthin (µg)	106.5 ± 147.8	44.7 (1.9–585.3) *	102.5 ± 125.4	39.4 (21.1–246.9)	0.827
	Lycopene (µg)	2099.5 ± 2457.3	1731.4 (0.0–13,411.9) *	2228.6 ± 2251.3	2182.4 (0.8–4502.7)	0.856
	Lutein + zeaxanthin (µg)	1787.9 ± 1913.5	1320.6 (289.0–13,221.3) *	615.9 ± 229.0	540.6 (434.0–873.1)	0.038
Intake per 1000 kcal	Vitamin A (µg retinol activity equivalent/1000 kcal)	689.1 ± 1336.4	366.8 (158.4–8947.0) *	505.6 ± 350.6	322.2 (284.6–909.8)	0.827
	Retinol (µg/1000 kcal)	383.7 ± 1164.1	193.5 (79.1–8640.2) *	302.2 ± 169.5	260.1 (157.8–488.8)	0.326
	β-carotene (µg/1000 kcal)	3085.3 ± 6584.0	1621.6 (61.3–45,899.7) *	2014.8 ± 1918.8	1674.5 (288.9–4081.0)	0.971
	α-carotene (µg/1000 kcal)	1031.0 ± 2707.1	409.8 (3.5–18,878.8) *	694.9 ± 1014.3	191.5 (30.7–1862.5)	0.689
	β-cryptoxanthin (µg/1000 kcal)	57.2 ± 102.8	15.8 (1.0–644.0) *	90.3 ± 132.5	17.8 (9.8–243.2)	0.636
	Lycopene (µg/1000 kcal)	969.9 ± 980.2	807.8 (0.0–3827.9) *	1815.1 ± 2324.6	1009.7 (0.4–4435.3)	0.636
	Lutein + zeaxanthin (µg/1000 kcal)	877.7 ± 706.4	723.7 (191.9–3505.7) *	357.7 ± 94.5	395.5 (250.1–427.5)	0.063

* Nonparametric distribution (verified using the Shapiro-Wilk test; *p* < 0.05).

**Table 5:** Summary table of human studies comparing vitamin A blood level or intake observed in inflammatory bowel disease and healthy individuals, as well as analysing associations between vitamin A blood level or intake and disease course or activity.

		Studied Group		
		Individuals with Inflammatory Bowel Disease	Individuals with Crohn’s Disease	Individuals with Ulcerative Colitis
Blood level	In comparison with level observed in healthy individuals	Lower [31,32]	Lower [33]	Lower [34]
	Association with disease course or activity	Associated [42,43]	Associated [18,41]	-
Intake	In comparison with level observed in healthy individuals	Higher intake of retinol [52]	-	Lower intake of vitamin A [28] and retinol [9]
	Association with disease course or activity	-	In case of exacerbation lower than in remission [22]	No variation between distal colitis, left colitis and pancolitis [8] In recurrent acute and chronic pouchitis lower than in normal pouch patients [9]
